# Breath Isoprene Sensor Based on Quartz-Enhanced Photoacoustic Spectroscopy

**DOI:** 10.3390/s25216732

**Published:** 2025-11-03

**Authors:** Fadia Abou Naoum, Diba Ayache, Tarek Seoudi, Daniel Andres Diaz-Thomas, Alexei Baranov, Fanny Pages, Julien Charensol, Eric Rosenkrantz, Meryem Aouadi, Michael Bahriz, Fares Gouzi, Aurore Vicet

**Affiliations:** 1 IES, Montpellier University, CNRS, 34090 Montpellier, France; fadia.abou-naoum@umontpellier.fr (F.A.N.); diba.ayache@umontpellier.fr (D.A.); tarek.seoudi@umontpellier.fr (T.S.); daniel.diaz@3-5lab.fr (D.A.D.-T.); alexei.baranov@umontpellier.fr (A.B.); fanny.pages1@umontpellier.fr (F.P.); julien.charensol@umontpellier.fr (J.C.); eric.rosenkrantz@umontpellier.fr (E.R.); michael.bahriz@umontpellier.fr (M.B.); 2 CHRU, 34000 Montpellier, France; meryem.aouadi@chu-montpellier.fr; 3 PhyMedExp, University of Montpellier, INSERM, CNRS, CHRU, 34095 Montpellier, France; f-gouzi@chu-montpellier.fr

**Keywords:** QEPAS, photoacoustics, isoprene, breath analysis

## Abstract

Isoprene, the most abundant endogenous hydrocarbon in human breath, is a promising biomarker for metabolic and cardiovascular diseases. In this paper, we present the detection of isoprene in exhaled breath using the off-beam Quartz-Enhanced Photoacoustic Spectroscopy (QEPAS) method. The sensor employs a homemade quantum cascade laser emitting at 11.03 μm. We use numerical simulations to evaluate the impact of interfering gases (CO_2_ and H_2_O) and optimize the laser modulation parameters. The limit of detection reached for 1 s acquisition time is close to 220 parts per billion in volume (ppbv) with a normalized noise equivalent absorption (NNEA) of $1.1\times 10^{-8}\;{\mathrm{cm}}^{-1}\;\cdot \;W\;\cdot \;{\mathrm{Hz}}^{-1/2}$. Breath measurements conducted on healthy volunteers reveal a significant increase in isoprene concentration from resting levels (~250–350 ppbv) to elevated levels (~450–650 ppbv) after moderate physical exercise.

## 1. Introduction

Isoprene (C_5_H_8_) was discovered in 1860 by C. G. Williams during the thermal decomposition of natural rubber, also known as latex or caoutchouc [1]. Industrially, isoprene is produced by the manufacturing of synthetic rubber [2]. At ambient temperature, isoprene is a colorless and volatile liquid with a specific petroleum odor that humans can smell from 5 parts per billion (ppbv) [3]. The definition of isoprene as a pollutant is not obvious as it is naturally produced by trees as a protection mechanism in case of exposure to moderate heat [4]. However, the reaction of this molecule with nitric oxides (NO_*x*_) produced by car exhausts could lead to the formation of ozone in the lower atmosphere. Whereas ozone is known to cause lung damage, direct exposure to isoprene is also suspected to cause irritation of the upper respiratory system and cancers. Physiologically, isoprene is the most abundant hydrocarbon produced endogenously by the body, and muscles have been identified as its main production sites [5,6]. In healthy individuals at rest, breath isoprene concentrations typically range from 100 to 250 ppbv. For instance, Mendis et al. [7] reported an average end-expiratory isoprene concentration of 172.4 ± 86.3 ppbv in healthy resting subjects, while different studies have shown a rapid increase—up to 800 ppbv—released within minutes after the beginning of physical exercise [8,9]. Pathologically, the increase of breath isoprene concentrations has been observed in cases of acute myocardial infarction [10].

Cardiovascular diseases (CVD) are the leading cause of death in the world; however, their diagnosis remains late, expensive, and invasive for patients. Being able to use the exhaled breath as a point of care tool would allow for rapid, easy, and non-invasive detection of CVD, but the detection technique must be appropriate for this application.

The “gold standard” method for trace gas analysis is Gas Chromatography (GC), often coupled with Mass Spectrometry (GC-MS), offering exceptional sensitivity in the part-per-trillion (pptv) range and high selectivity. For measurements of trace gases in a complex matrix, a pre-concentration step is usually performed [11]. GC-MS has been successfully employed in various isoprene detection applications, including analysis of plant emissions [12] and exhaled human breath [13,14]. For example, Schulz et al. [15] reported isoprene levels in breath samples of 170 and 990 ppbv in healthy subjects at rest. However, the large size, cost, and operational complexity of GC–MS, including its need for extensive sample preparation, make it unsuitable for clinical applications.

Selected ion flow tube mass spectrometry (SIFT-MS) offers an alternative approach that enables direct, real-time breath analysis without pre-concentration. Turner et al. achieved a limit of detection (LOD) of 5 ppbv for isoprene and reported alveolar concentrations from 0 to 474 ppbv in healthy volunteers [16]. Furthermore, they observed that within 5 s of initiating physical exercise, resting isoprene concentrations of 199–321 ppbv increased by a factor of 1.6–2.3 (to 349–504 ppbv) [16]. Despite these capabilities, SIFT-MS systems remain relatively large and expensive and require skilled operators.

Another family of gas sensors, namely optical sensors, presents excellent selectivity and very good sensitivity, challenging that of the GC-MS. In several applications, these sensors have been employed for real-time or almost real-time detection. For instance, Cavity Ring Down Spectroscopy (CRDS) in the ultraviolet (UV) domain has been implemented for the investigation of isoprene in the breath of lung cancer patients, achieving an LOD of 0.47 ppbv and measuring concentrations between 51.4 and 209 ppbv [17]. Infrared (IR) spectroscopy in the $\nu_{26}$ and $\nu_{17}$ bands of isoprene near 992 cm^−1^ has also been demonstrated using multipass cells [18], achieving an LOD of 3.2 ppbv with an averaging time of 9 s. The same mid-IR region was targeted using photoacoustic spectroscopy (PAS) using a 600 mW CO_2_ laser, and the setup performances reached a LOD close to 400 pptv [19]. More recently, Pangerl et al. introduced a PAS system with an interband cascade laser (ICL) for breath isoprene detection, reporting an LOD of 26.9 ppbv and a normalized noise equivalent absorption (NNEA) coefficient of $1.1\times 10^{-8}\;W\;{\mathrm{cm}}^{-1}\;{\mathrm{Hz}}^{-1/2}$ [20].

In this paper, we present the first use of Quartz-Enhanced Photoacoustic Spectroscopy (QEPAS) for the detection of breath isoprene in healthy volunteers both at rest and immediately after the start of exercise, in order to measure higher levels of this hydrocarbon. Compared to the previously mentioned techniques, QEPAS has high potential for compactness, as the detected signal is proportional to the power of the infrared source and not to a specific optical path. It can lead to real-time measurements since no long integration time or pre-concentrators are needed, unlike GC-MS techniques. QEPAS relies on photoacoustic spectroscopy principles but uses a quartz tuning fork (QTF) as transducer of the photoacoustic signal. When the acoustic wave, generated at a frequency $f_{\mathrm{QTF}}$, meets the prongs of the QTF, the resulting in-plane mechanical movements are converted into an electrical signal by piezoelectric effect. This signal is proportional to the concentration of the absorbing gas present in the medium. To prevent and avoid photothermal perturbations generated by the laser light hitting the prongs of the QTF, an off-beam configuration is adopted [21]. In this case, the light of the laser is focused inside a T-shaped acoustic cavity instead of being focused between the QTF prongs. The acoustic cavity is geometrically designed to enhance the confinement of the acoustic wave and thus increase the sensor sensitivity. Together, these features enable a compact, sensitive, and real-time-capable breath analyzer. Note, however, that in this study we perform an offline analysis of end-tidal breath samples rather than real-time online analysis.

## 2. Experimental Setup

The sensor uses QEPAS in an off-beam configuration and a distributed-feedback (DFB) quantum cascade laser (QCL) emitting at 11.03 μm (906.3 cm^−1^), developed and fabricated at the University of Montpellier (UM) to target the $\nu_{27}$ band of isoprene.

**Figure 1:**
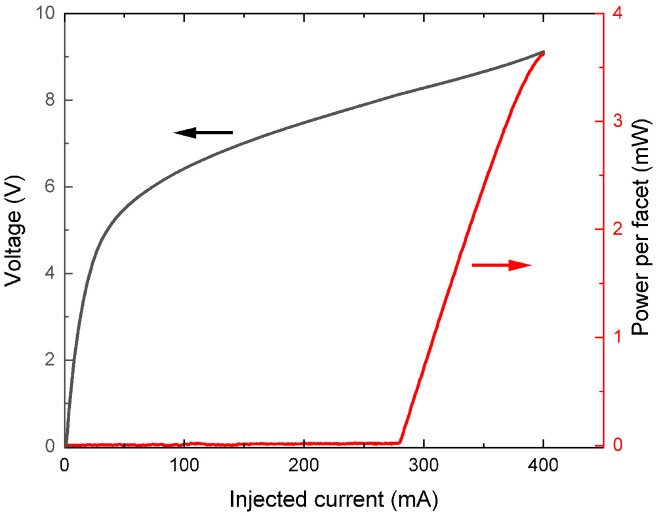
Laser output power (left axis) and measured voltage (right axis) as a function of the injected current for the DFB-QCL operating at 2 °C in a continuous-wave regime. The power-meter is placed after a KBr window and a black diamond focusing lens of 90% and 95% transmissions, respectively.

The QCL, fabricated by molecular beam epitaxy in the InAs/AlSb material family [22], operates in continuous-wave (CW) regime over the temperature range −10 °C to 5 °C, with a side mode suppression ratio (SMSR) of 20 dB. Figure 1 presents its emitted power and voltage as a function of the injected current in the continuous wave regime at 2 °C. Further measurements are performed using the laser operating at this temperature, where the threshold current value is 280 mA, and the maximum output power reaches 3.6 mW at 400 mA.

**Figure 2:**
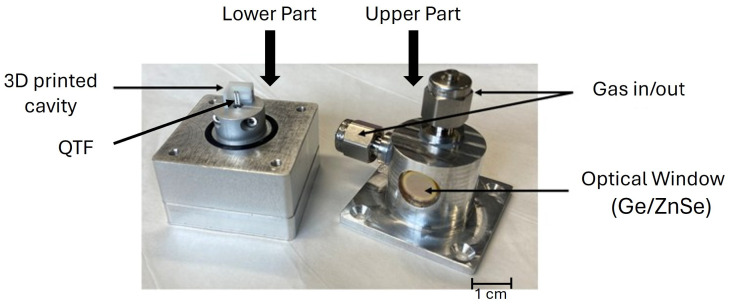
Disassembled gas cell with reduced volume. Compared to [23] where the volume of the gas cell was around 60 mL, the present one is close to 2 mL.

The laser chip is enclosed in a homemade mounting and connected to a current driver and temperature controller (Thorlabs ITC4005 QCL). The temperature regulation is performed with a PT100 temperature sensor and a Peltier cooler. A water-cooling system is used for heat dissipation of the Peltier cooler at low temperatures. The laser mount is closed under dry nitrogen to avoid any water condensation on the chip. The emitted beam of the laser passes through a KBr window (transmission T=90%) and is collimated into the gas cell by the mean of a black diamond lens (Thorlabs C028TME-F) of focal length f=5.95 mm. The window of the gas cell (transmission T=95% at 11 μm) is made of coated germanium (3–12 μm) to minimize the laser power losses occurring at this stage. Into the gas cell, the laser beam is focused inside a 3D printed acoustic cavity designed and adjusted to reach the resonance frequency of a commercial tuning fork ($f_{\mathrm{QTF}}=32,742.3$ Hz). The experimental QEPAS setup follows the design of [23], with a more compact gas cell (Figure 2), which reduces the analyzed volume and the reaction time of the setup.

## 3. Spectral Simulations and Modulation Techniques

### 3.1. Spectral Simulations

**Figure 3:**
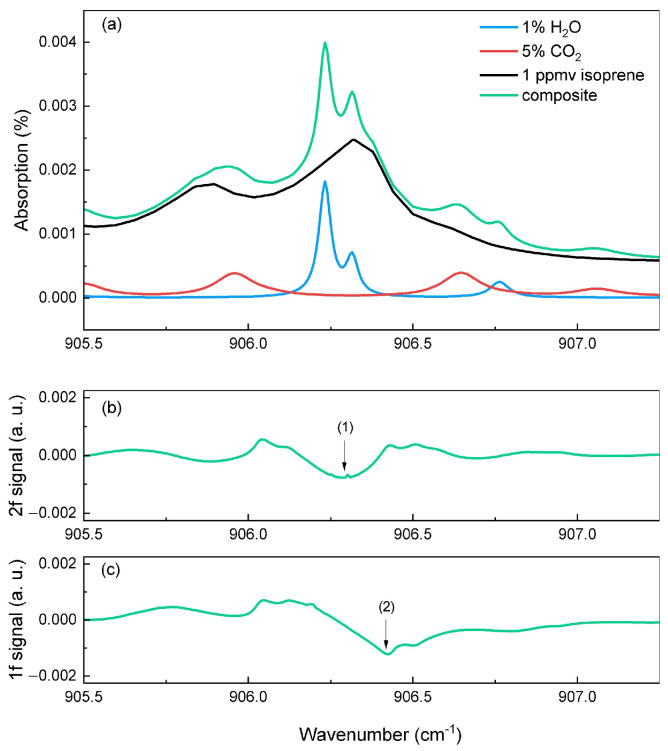
(**a**) Simulated absorption lines of isoprene, H_2_O, and CO_2_ from HITRAN database [24] and their composite for a typical breath mixture [25] (1 part per million (ppmv) isoprene, room ambient value (1%) for H_2_O and 5% CO_2_) over a 1 cm path length. (**b**) Simulated second-harmonic (2f) QEPAS signal of the composite. (**c**) Simulated first-harmonic (1f) QEPAS signal of the composite. The 1f and 2f signals represent the first and second derivatives of the composite absorption profile.

To measure isoprene in breath, the laser is tuned to the target absorption line at 11.03 μm and operated at 2 °C in continuous wave regime. Using the cross section data from the HITRAN database [24], we simulated the absorption spectra of the main gases found in a typical exhaled breath mixture (isoprene, H_2_O and CO_2_) [25,26]. While CO_2_ and H_2_O can reach concentrations of about 5% in exhaled air, the use of a Nafion tube (BE series Moisture Echangers) in our measurement setup significantly reduces the H_2_O concentrations to around 1%. This tube, provided by the Permapure company, is made of Nafion membranes that effectively decrease humidity in a gas mixture. Nafion is a polymer of Teflon with a fluocarbon and sulfonic acid side chains. When a humidified air flows through the Permapure tube, the water is absorbed by the sulfonic group. Then, the water diffuses to the environment creating an equilibrium between the mixture and the ambient level of humidity (around 1%). Therefore, these values were used in the simulations to accurately reflect the measurement conditions. Figure 3a shows the absorption lines of isoprene (black), CO_2_ (red) and H_2_O (blue) at concentrations of 1 ppmv, 5%, and 1%, respectively, over a 1 cm path length at ambient temperature and pressure conditions. The green curve represents the composite spectrum resulting from the sum of these three gases, showing the spectral overlap between them. The strongest peak of this composite profile, at 906.3 cm^−1^, results from the combined contributions of isoprene and H_2_O. However, the slope of this peak at 906.4 cm^−1^ follows the absorption profile of isoprene without interference with other gases. The contribution of CO_2_ is about five times weaker than that of water, making it negligible and far from the target region.

The emitted wavelength of the laser is modulated by adding a sinusoidal high frequency signal to a slow scan across the absorption line. In QEPAS, this high frequency corresponds to a harmonic of the QTF fundamental frequency, $f_{\mathrm{QTF}}$. According to Arndt’s theory [27], the resulting signal is proportional to the derivative of the absorption line: by modulating the wavelength at $f=f_{\mathrm{QTF}}$ (1f) or $f=f_{\mathrm{QTF}}/2$ (2f), the detected signal becomes proportional to the first or second derivative of the absorption line, respectively. Arndt’s theory applies under the assumption of wavelength small modulation amplitudes, corresponding to $\Delta \nu \ll 2\;\Delta \nu_{\mathrm{linewidth}}$, where Δν is the laser frequency excursion and $\Delta \nu_{\mathrm{linewidth}}$ denotes the full width at half maximum (FWHM) of the absorption line.

The modulated frequency can be expressed as $\nu \left(t\right)=\nu_{c}+\Delta \nu \sin\left(\omega t\right)$, where Δν is the modulation amplitude. We consider that when the laser is modulated, the emitted power remains constant. The absorption coefficient of the gas is calculated by expansion of the absorption function in a Fourier series [28,29]. Figure 3b,c display the simulated 1f and 2f QEPAS signals corresponding to the first and second derivatives of the composite absorption spectrum. These simulations were done by using a small modulation amplitude Δν corresponding to a Δσ = 0.02 cm^−1^ in wavenumber.

The experimentally measured signal of exhaled breath at the selected wavelength (11.03 μm) corresponds to the 1f or 2f response of the composite spectrum, which includes contributions from three gases: isoprene, H_2_O, and CO_2_. Therefore, it is essential to evaluate how much the interfering gases (H_2_O and CO_2_) affect or distort the measured signal in order to reliably extract the isoprene concentration and get rid of this background. Figure 3b shows the 2f signal, which follows the second derivative of the absorption spectrum and produces a negative lobe (1) centered on the absorption maximum (around 906.3 cm^−1^), where both water and isoprene absorb. In Figure 3c, the 1f signal exhibits the first derivative shape, crossing zero at the absorption maximum. Its negative lobe (2) near 906.4 cm^−1^ coincides with the slope of the composite spectrum that comes mainly from isoprene without interfering gases. These simulations show that 2f detection is affected by water interference, while 1f detection provides more selective response to isoprene. We will confirm this assumption later in Section 3.3.

### 3.2. Optimization and Experimental Conditions

**Figure 4:**
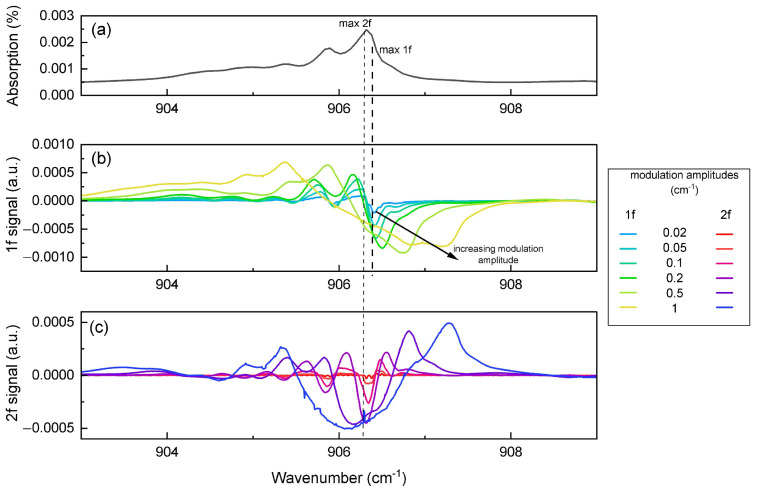
Absorption spectrum (**a**) and corresponding 1f (**b**) and 2f (**c**) QEPAS signals of pure isoprene for different modulation amplitudes: 0.02, 0.05, 0.1, 0.2, 0.5, and 1 cm^−1^. Distortion increases at higher modulation amplitudes.

The modulation amplitude must be adjusted to optimize the sensor performances. As presented in Figure 4a, the isoprene absorption spectrum is broad. Therefore, the optimum modulation amplitude does not behave as presented in wavelength modulation spectroscopy (WMS) theory [27,30,31]. Figure 4b,c present the simulated 1f and 2f QEPAS signals of pure isoprene, respectively, at different modulation amplitudes: 0.02, 0.05, 0.1, 0.2, 0.5, and 1 cm^−1^. At low amplitudes (0.02 and 0.05 cm^−1^), the QEPAS signal is weaker, while large amplitudes (0.5 or 1 cm^−1^) induce significant signal deformations.

In the following experimental study, QEPAS signals are obtained for an isoprene concentration of 50 ppmv. The laser is driven using a slow current ramp to which is added a high frequency (32,768 kHz) sinewave of 25 mA modulation amplitude, corresponding to 0.1 cm^−1^, for WMS signal generation. The lock-in amplifier (EG&G 7260) time constant is set to 100 ms. The power of the laser, measured after the acoustic cavity, at 344 mA, ranges around 1.2 mW.

**Figure 5:**
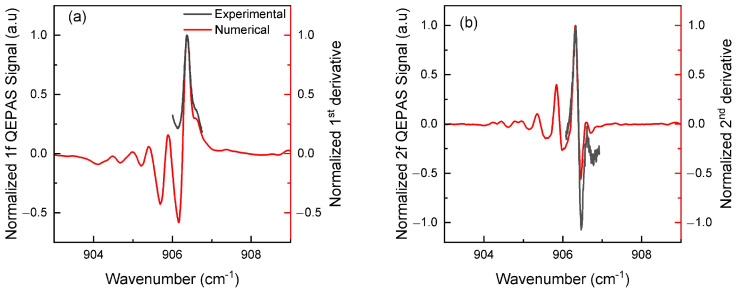
Experimental (black) and numerical (red) QEPAS signals of 50 ppmv of diluted isoprene in nitrogen as a function of the wavenumber: (**a**) 1f and (**b**) 2f signals using 0.1 cm^−1^ as modulation amplitude. The simulated signals, obtained at atmospheric pressure and 300 K, are inverted for comparison purposes.

Figure 5 compares the measured (black) and numerically simulated (red) QEPAS signals of isoprene for both 1f and 2f components, using a modulation amplitude of 0.1 cm^−1^. Both harmonics (1f and 2f) show good agreement between the measurements and simulations in terms of shape and width. The first lobe of the 1f signal appears around 906.4 cm^−1^, while for the 2f signal, the secondary (negative) lobe is located near 906.5 cm^−1^ and the main (positive) lobe around 906.3 cm^−1^. Other lobes clearly seen in the simulations beyond the main lobes are not visible experimentally, mainly due to the laser’s current limitations (Figure 1).

### 3.3. Isoprene in Breath: Numerical Interpretations

**Figure 6:**
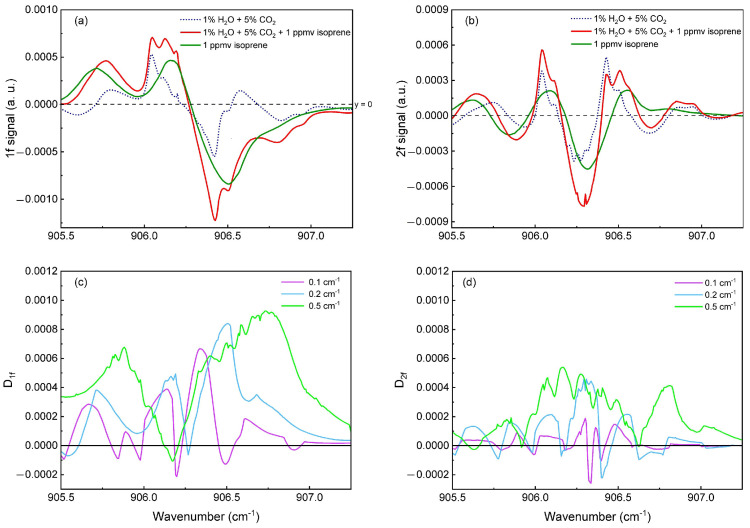
(**a**) Simulated 1f QEPAS signals for various gas mixtures. (**b**) Simulated 2f QEPAS signals for various gas mixtures. (**c**) Difference parameter $D_{1f}$, defined as the subtraction of the background from the total signal, plotted as a function of wavenumber for different modulation amplitudes (0.1, 0.2, and 0.5 cm^−1^). (**d**) Equivalent difference parameter $D_{2f}$ for 2f signals.

The modulation amplitude was previously studied on the absorption spectrum of pure isoprene. In this section, we extend the analysis by performing additional numerical simulations to evaluate the influence of background gases (H_2_O and CO_2_) on the QEPAS signal. The goal is to understand how these interfering species affect the signal amplitude and lineshape, and to assess the robustness of isoprene detection under realistic breath conditions. Figure 6a shows the simulated 1f signals for different gas mixtures at a fixed modulation amplitude of 0.2 cm^−1^. The dotted blue curve corresponds to the signal of H_2_O (1%) and CO_2_ (5%) alone, representing the background contribution from breath gases. The green curve shows the 1f signal of pure 1 ppmv isoprene. The red curve represents the 1f signal of the full mixture (H_2_O + CO_2_ + isoprene at 1 ppmv). Figure 6b shows equivalent simulations for the 2f signal. These simulations allow us to see how the presence of background gases modifies the shape and amplitude of the signal.

To isolate the isoprene contribution, we define a difference parameter D, calculated as

D_1*f*,2*f*_ = 1f,2f(H_2_O + CO_2_ + isoprene) − 1f,2f(H_2_O + CO_2_) (1)

This parameter *D* represents the contribution of isoprene to the total QEPAS signal by subtracting the background response of H_2_O and CO_2_. A larger D value indicates that the isoprene response is more distinct and less affected by interfering gases, while smaller values suggest stronger interference. We calculated D for several modulation amplitudes (0.1, 0.2, and 0.5 cm^−1^) in both 1f and 2f modes. The results, shown in Figure 6c,d, plot D as a function of wavelength. These plots allow the identification of the (i) modulation amplitude and (ii) wavelength regions where the isoprene sigature is maximized relative to the background.

The results demonstrate that D values are consistently higher in the 1f mode (Figure 6c) than in the 2f mode (Figure 6d), indicating that 1f detection is less affected by H_2_O and CO_2_. This difference arises because the maximum of the 1f isoprene signal is spectrally shifted from that of H_2_O and CO_2_, while the 2f isoprene peak overlaps with that of water, which reduces its distinctiveness. Therefore, operating in the 1f modulation is more advantageous to minimize the influence of interfering gases for the detection of low isoprene concentrations in exhaled breath.

The simulations also show that both the magnitude and the spectral shape of D strongly depend on the modulation amplitude. While larger amplitudes (0.5 cm^−1^) increase the magnitude of D, they also broaden the derivative features and degrade spectral resolution due to over-modulation. A modulation amplitude between 0.1 cm^−1^ and 0.2 cm^−1^ appears to provide the best compromise, maximizing D while preserving the spectral shape of the signal.

Based on these optimized conditions, we estimated the limit concentration of isoprene in H_2_O and CO_2_ based on a signal-to-noise ratio (SNR) of 1. This corresponds to the lowest concentration of isoprene that can be detected with our setup. The concentration giving the same amplitude signal as the H_2_O + CO_2_ corresponds to 90 ppbv.

## 4. Sensor Calibration

**Figure 7:**
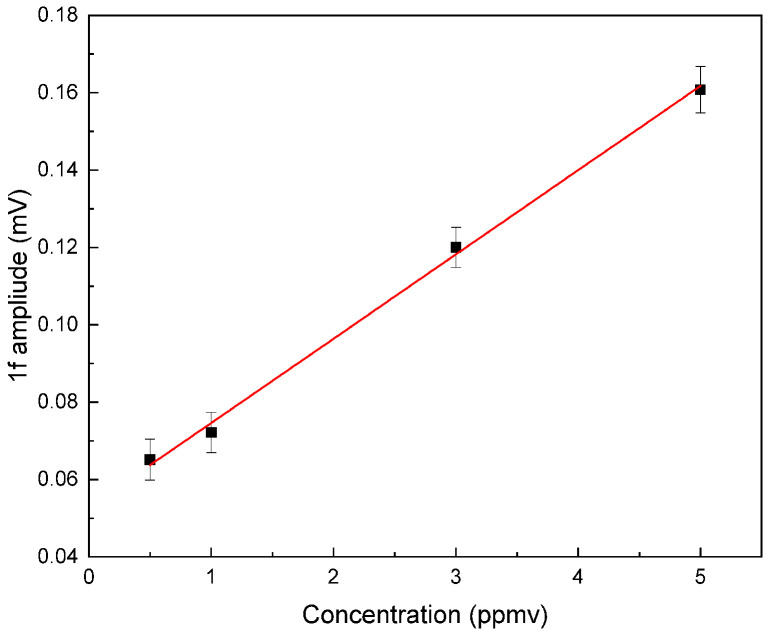
1f QEPAS signal amplitude observed for 0.5, 1, 3, and 5 ppmv of pure isoprene in nitrogen. The time constant of the lock-in amplifier is set to 500 ms and the laser current is fixed at 343 mA. The error bars correspond to the measured noise level with standard deviation, evaluated as being close to ±0.005 mV. The red line represents the linear fit, and the black circles correspond to the experimental data points.

Following the selection of the 1f detection mode to minimize water effect and a modulation amplitude of 25 mA, we proceed to evaluate the limit of detection of the sensor as well as its linearity through gas calibrations measurements. Isoprene concentrations ranging from 0.5 to 5 ppmv are prepared in Tedlar bags by diluting a 50 ppmv calibrated gas bottle using a gas mixing system (GasMix-2 Aiolos). This system, equipped with mass flowmeters, provides controlled dilutions in dry nitrogen at atmospheric pressure with a constant flow of 250 mL/min. The measured 1f signal amplitude as well as the associated standard deviation are presented in Figure 7. The lock-in time constant is set to 500 ms. A non-zero background of 0.05 mV (no gas) is observed in 1f configuration as the sensor is most sensitive to photothermal induced and cavity walls noises.

**Figure 8:**
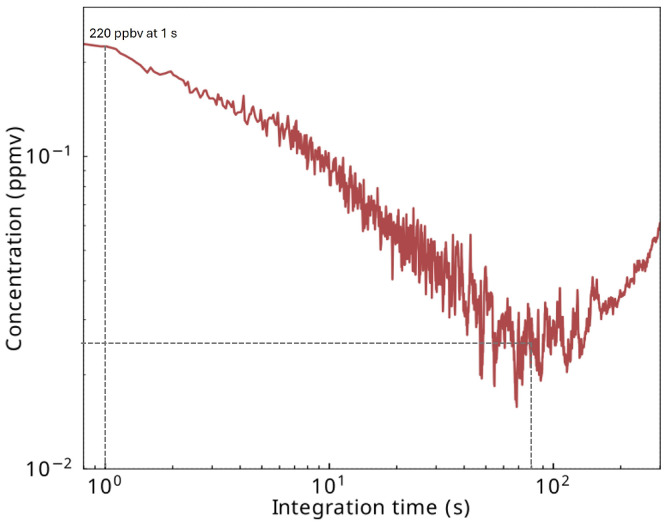
Allan–Werle deviation obtained for 1f QEPAS signal for an isoprene concentration of 1 ppmv. The lock-in time constant is 200 ms. The laser is operated at 2 °C and 343 mA.

Allan–Werle [32] deviation was performed in 1f detection. This statistical tool is used to quantify the performances of a sensor in terms of limit of detection for a given integration time. The extracted information is further used to calculate the normalized noise equivalent absorption (NNEA) of the sensor. Allan–Werle deviation was recorded using an isoprene concentration of 1 ppmv with a lock-in amplifier time constant of 200 ms. The modulation amplitude in 1f was set to 25 mA, and the laser was operated at 343 mA. The results are presented in Figure 8.

The limit of detection (LOD) of pure isoprene in $N_{2}$ for a 1 s acquisition time (1σ) is approximately 220 ppbv, and the optimum acquisition time is found around 80 s giving a minimum LOD of 27 ppbv, represented with the dashed lines on Figure 8. These LODs demonstrate the capability of our sensor for measuring isoprene concentrations level found in the breath. During a physical exercise, measurements performed on the exhaled breath of healthy volunteers have shown levels ranging from 200 ppbv to 800 ppbv [8]. In the following part, the designed sensor is used for an offline analysis of exhaled isoprene during a physical exercise.

## 5. Breath Measurements

The exhaled breath is almost saturated with water vapor, typically exceeding 80% relative humidity (RH) which corresponds to a concentration of about 5% in volume, at 33 °C breath temperature and ambient pressure. In addition to being a potential interferent, water is known to influence the photoacoustic signal [33]. To mitigate this effect during breath measurements, the humidity is stabilized at a level close to the experimental room ambient humidity (40% RH at 20 °C ≈ 9300 ppmv) using a 30 cm long Nafion tube ($BE^{TM}$ series Moisture Exchangers). Consequently, by working in an air conditioned room, the measured signal is not affected by any potential humidity variation.

**Figure 9:**
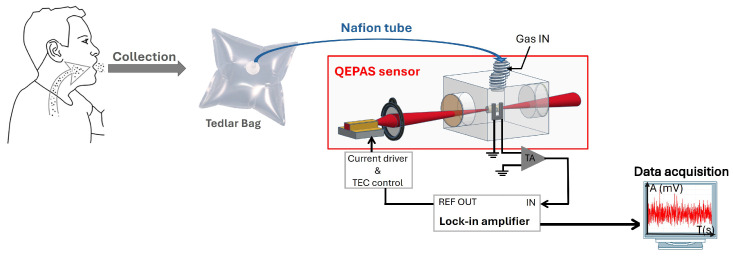
Experimental setup for the measurement of isoprene in exhaled breath.

To perform breath measurements, the subject inhales fully and then exhales slowly and steadily until emptying the lungs to capture the sample breath, containing a major part of the alveolar air. This end-tidal volume corresponds to the air from deep lungs where gas exchange with blood occurs and reflects the systemic (blood) levels of volatile compounds. The experimental setup is presented in Figure 9. The exhaled sample is collected in a 5 L Tedlar bag, which is inert to the analyzed gases. This volume is then immediately directed into the gas cell at a flow rate of 500 mL min^−1^ through the Nafion tube. Finally, the breath sample is analyzed by the QEPAS sensor to determine the isoprene concentration. The demodulated QEPAS signal is recorded via a homemade python graphical user interface for data analysis.

Before each measurement, the gas cell was flushed with nitrogen to determine the baseline signal. Then, we recorded the signal given by ambient room air to determine the continuous H_2_O signal in the environment. Since H_2_O is also found in the exhaled sample breath and affects the measured 1f signal (Section 3), its contribution must be considered in the analysis. We assume that the ambient air signal, corresponding to about 1% H_2_O, was equivalent to the H_2_O concentration in breath after passing through the Nafion tube. In this way, the ambient air signal was used as a reference for the H_2_O contribution in the breath sample. This recorded air signal was then subtracted from the sample breath signal to remove the H_2_O contribution and accurately isolate the isoprene concentration. Consequently, the values of exhaled breath reported in this study reflect the pure endogenous isoprene concentrations.

The measurement protocol consisted of four successive acquisition phases, each lasting 200 s:

Phase 1—Nitrogen flush.
Phase 2—Resting breath sample: After the volunteer remained seated and relaxed for several minutes to stabilize metabolic activity and allow the exhaled isoprene concentration to reach a steady state, a breath sample was collected in a Tedlar bag at atmospheric pressure and analyzed using the QEPAS setup.
Phase 3—Nitrogen flush: This was performed to eliminate any residual gases from previous measurements.
Phase 4—Exercise breath sample: A second breath sample was collected one minute after moderate physical activity (cycling on a stationary bicycle).

**Figure 10:**
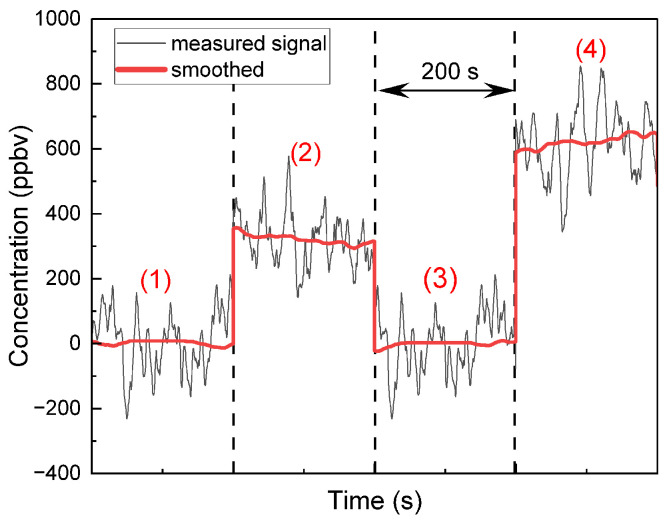
Measured isoprene concentrations from one *single* healthy male volunteer (30 years, 75 kg bodyweight) under five conditions, each lasting 200 s: (1) nitrogen baseline, (2) breath at rest, (3) nitrogen, (4) breath during exercise. The black curve represents the raw signal and is used to calculate the signal’s noise at each phase, and the red line shows the smoothed trend for each phase.

**Figure 11:**
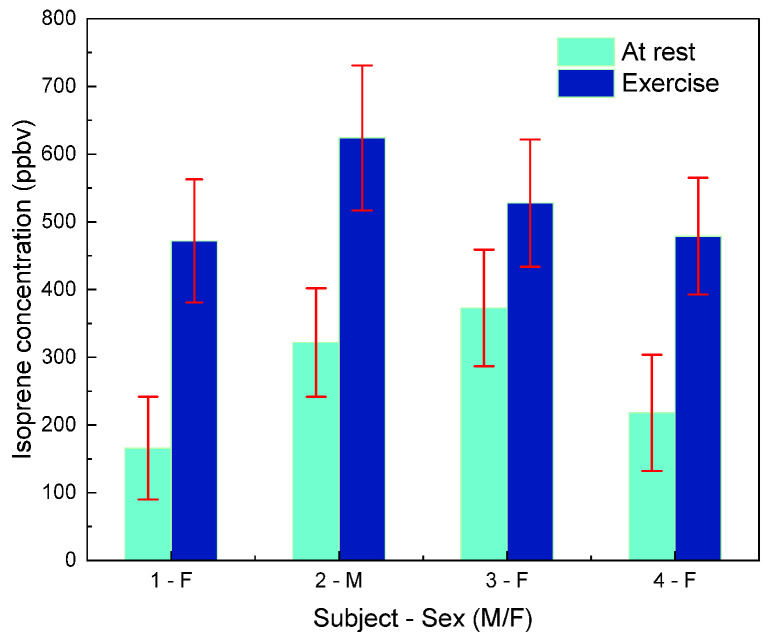
Breath isoprene concentrations measured by QEPAS sensor for four subjects at rest and during cycling exercise. Errors bars in red represent the noise of the measured signal.

These four phases are represented in Figure 10 for one healthy, non-smoking volunteer. The red line represents the smoothed mean signal over each 200 s acquisition interval. In Phase 2 (resting breath), the isoprene concentration reaches an average of 350 ppbv. During Phase 4 (exercise breath), the isoprene concentration increases significantly, reaching up to 650 ppbv. The entire procedure was repeated under the same conditions for four different volunteers (one male, 30 years old; three females, 23, 25, and 50 years old), each tested at rest and immediately at the start of physical activity. The values of isoprene at rest and during cycling, are represented in the following histogram (Figure 11).

In our measurements, peaks of isoprene ranging from 450 ppbv to 650 ppbv were detected during exercise, compared to lower values between 250 ppbv and 350 ppbv at rest. This increase of the isoprene concentration in the exhaled breath at the beginning of the effort can be attributed to its endogenous production in muscle tissue. During a physical exercise the blood flows significantly through the muscles and washes out the isoprene that is transported in the systemic circulation until being evacuated from the body during the breathing process.

## 6. Conclusions

This paper presents the first demonstration of isoprene detection using Quartz-Enhanced Photoacoustic Spectroscopy (QEPAS). The system has been developed using a prototype QCL designed and fabricated at Montpellier University. The performance of the sensor leads to an NNEA of about $1.1\times 10^{-8}\;{\mathrm{cm}}^{-1}\;\cdot \;W\;\cdot \;{\mathrm{Hz}}^{-1/2}$, reaching the state of the art for QEPAS sensors. The developed sensor has allowed measurement in the breath of healthy volunteers during physical exercise, where a significant increase in isoprene levels was observed. This designed system constitutes a first step in the diagnosis and analysis of exhaled breath using QEPAS sensors for diseases influencing the endogenous isoprene levels, such as heart diseases. It could lead to innovative non-invasive point of care tools for diagnosis and telediagnosis. 

## Data Availability

Data underlying the results presented in this paper are not publicly available at this time but may be obtained from the authors upon reasonable request.
